# Usability of novel major TraumaApp for digital data collection

**DOI:** 10.1186/s12873-022-00578-9

**Published:** 2022-03-12

**Authors:** Joanna Butler, Evan Wright, Lucy Longbottom, Alan S. Whitelaw, Kevin Thomson, Malcolm W. G. Gordon, David J. Lowe

**Affiliations:** 1 Emergency Department, Queen Elizabeth University Hospital, Glasgow, UK; 2 University of Glasgow, Glasgow, UK; 3 Plymouth Medical School, Plymouth, UK

**Keywords:** Trauma, Digital, Data collection, System usability score, TraumaApp, Clinical decision support

## Abstract

**Background:**

Delivery of major trauma care is complex and often fast paced. Clear and comprehensive documentation is paramount to support effective communication during complex clinical care episodes, and to allow collection of data for audit, research and continuous improvement. Clinical events are typically recorded on paper-based records that are developed for individual centres or systems. As one of the priorities laid out by the Scottish Trauma Network project was to develop an electronic data collection system, the TraumaApp was created as a data collection tool for major trauma that could be adopted worldwide.

**Methods:**

The study was performed as a service evaluation based at the Queen Elizabeth University Hospital Emergency Department. Fifty staff members were recruited in pairs and listened to five paired major trauma standby and handover recordings. Participants were randomised to input data to the TraumaApp and one into the existing paper proforma. The time taken to input data add into was measured, along with time for clarifications and any errors made. Those using the app completed a System Usability Score.

**Results:**

No statistically significant difference was demonstrated between times taken for data entry for the digital and paper documentation, apart from the Case 5 Handover (*p* < 0.05). Case 1 showed a significantly higher time for clarifications and number of errors with digital data collection (*p* = 0.01 and *p* = 1.79E-05 respectively). There were no other differences between data for the app and the proforma. The mean System Usability score for this cohort was 75 out of 100, with a standard deviation of 17 (rounded to nearest integer).

**Conclusion:**

Digital real-time recording of clinical events using a tool such as the TraumaApp is comparable to completion of paper proforma. The System Usability Score for the TraumaApp was above the internationally validated standard of acceptable usability. There was no evidence of improvement in use over time or familiarity, most likely due to the brevity of the assessments and the refined user interface. This would benefit from further research, exploring data completeness and a potential mixed methods approach to explore training requirements for use of the TraumaApp.

**Supplementary Information:**

The online version contains supplementary material available at 10.1186/s12873-022-00578-9.

## Background

Trauma contributes to 8% of all deaths worldwide [1]. It is the leading cause of death in Western countries for those under 40 years old [2]. Inclusive trauma systems have been shown to improve outcomes in major trauma [3]. The Scottish Trauma Network (STN) was established to coordinate care between the Scottish Ambulance Service, local trauma units with the creation four newly created of four Major Trauma Centres (MTCs) [2]. Development of the network has provided the opportunity to standardise processes with the need for granular data to monitor impact of this investment on patient outcomes. Clear and comprehensive documentation is paramount to enable effective communication between teams [4], and to allow collection of data for audit, enabling systems to systematically improve clinical care [5].

The National Institute for Health and Care Excellence guidelines lay out consensus criteria for complete trauma documentation, including Standby and Handover information, A to E assessment (complete primary assessment of a trauma patient in critical order as per Advanced Trauma Life Support guidance) [6] and interventions recorded contemporaneously by a designated member of the trauma team [7].

Similar to the majority of centres worldwide, the Queen Elizabeth University Hospital (QEUH) in Glasgow trauma case management is currently documented on a paper proforma. While documents across trauma systems capture similar key data there is considerable variation and compliance with completion is limited [8]. One of the priorities laid out by the Scottish Trauma Network (STN0) in 2019/20 was to develop an electronic data collection system, allowing easier and more accurate data collection [5].

Electronic documentation has consistently been shown to improve the quality and utility of documentation by reducing errors, omissions [9, 10] and length of stay in the Emergency Department (ED) [11, 12]. Baumann et al. performed a systematic review which showed that, initially, documentation time was slower following implementation of an electronic documentation system. However, as familiarity increased the documentation time decreased again [13]. The TraumaApp was developed as a collaboration between the EmQuire research group, Scottish Trauma Network and Daysix, a digital transformation company. The work was funded by InnovateUK (Project 104,540) to create a data collection tool for major trauma that could be adopted worldwide. During the two-year project, extensive co-design involving a range of trauma clinicians enabled rapid wireframing and prototyping to refine the tool. The data dictionary was based on current Scottish Trauma Audit Group and Trauma Audit and Research Network (TARN) data reporting and was aligned to international reporting standards [5, 14, 15].

## Methods

The aim was to compare the feasibility of using a digital data collection tool (TraumaApp) against the paper trauma proforma used in the ED at the QEUH specifically,

- The time taken to input information and the number of mistakes or clarifications required.
- Assess usability of the TraumaApp

Secondary aims included assessment of improvement in digital tool use over time, suggesting familiarity, and gaining qualitative feedback on app use.

The study was performed as a service evaluation based at the Queen Elizabeth University Hospital ED. This tertiary ED sees approximately 110,000 > 16-year-old presentations per year and will be officially designated a MTC in August, 2021. The department is currently staffed by 30 consultants, 55 training grade doctors and 122 nurses. All staff types may be expected to use the TraumaApp including new roles such as trauma co-ordinators.

Fifty ED Staff members were recruited in pairs (Table 1). They were consented and, in each pair, one participant entered data into the TraumaApp and one into the existing paper proforma. Participants listened to five paired Standby and Handover recordings (Additional file 1) in sequence, with the cases in random order (Table 2) using a random number generator. Staff were familiar with the paper documentation from previous clinical exposure and departmental educational sessions. Participants were timed to assess how long it took to input the data after each recording was completed. Additionally, the time taken for clarifications was also recorded. Finally, the proforma and the app were assessed against a “template” and any mistakes and omissions were counted.

**Table 1:** Participant Demographics

**Demographics**	Digital *n* = 24 (% of digital)	Paper *n* = 24 (% of paper)	Total *n* = 48 (% of total)
**Job Role**			
Consultant	4 (16.6%)	3 (12.5%)	7 (14.6%)
Junior Doctor	14 (58.3%)	16 (66.6%)	30 (62.5%)
ACNP^1^	0	1 (4.2%)	1 (2.1%)
Nurse	4 (16.6%)	3 (12.5%)	7 (14.6%)
Major Trauma Coordinator	2 (8.3%)	0	2 (4.2%)
Student Nurse	0	1 (4.2%)	1 (2.1%)
**Years in ED (mean)**	5.57	5.21	5.39
**Own Tablet**	16 (68%)	18 (76%)	34 (70.8%)
Apple	16 (68%)	16 (68%)	32 (68%)
Other	0	2 (8.3%)	2 (4.2%)
**Own Smartphone**	24 (100%)	24 (100%)	48 (100%)
Apple (TraumaApp is iOS tool)	17 (70.8%)	14 (58.3%)	31 (64.6%)
Other	7 (29.2%)	10 (41.7%)	17 (35.4%)
**Confidence with digital data input**	4.38	4.79	4.58
**Frequency of use**	4.46	4.5	4.48

*ACNP* Acute care nurse practitioner

**Table 2:** Order in which cases were played

	**Case 1**	**Case 2**	**Case 3**	**Case 4**	**Case 5**
**Standby duration (secs)**	41	55	21	41	25
**Handover duration (secs)**	43	36	58	76	50
**Order**					
1st	7	4	5	1	7
2nd	4	5	5	9	1
3rd	1	4	5	6	8
4th	6	6	4	4	4
5th	6	5	5	4	4

**Fig. 1:**
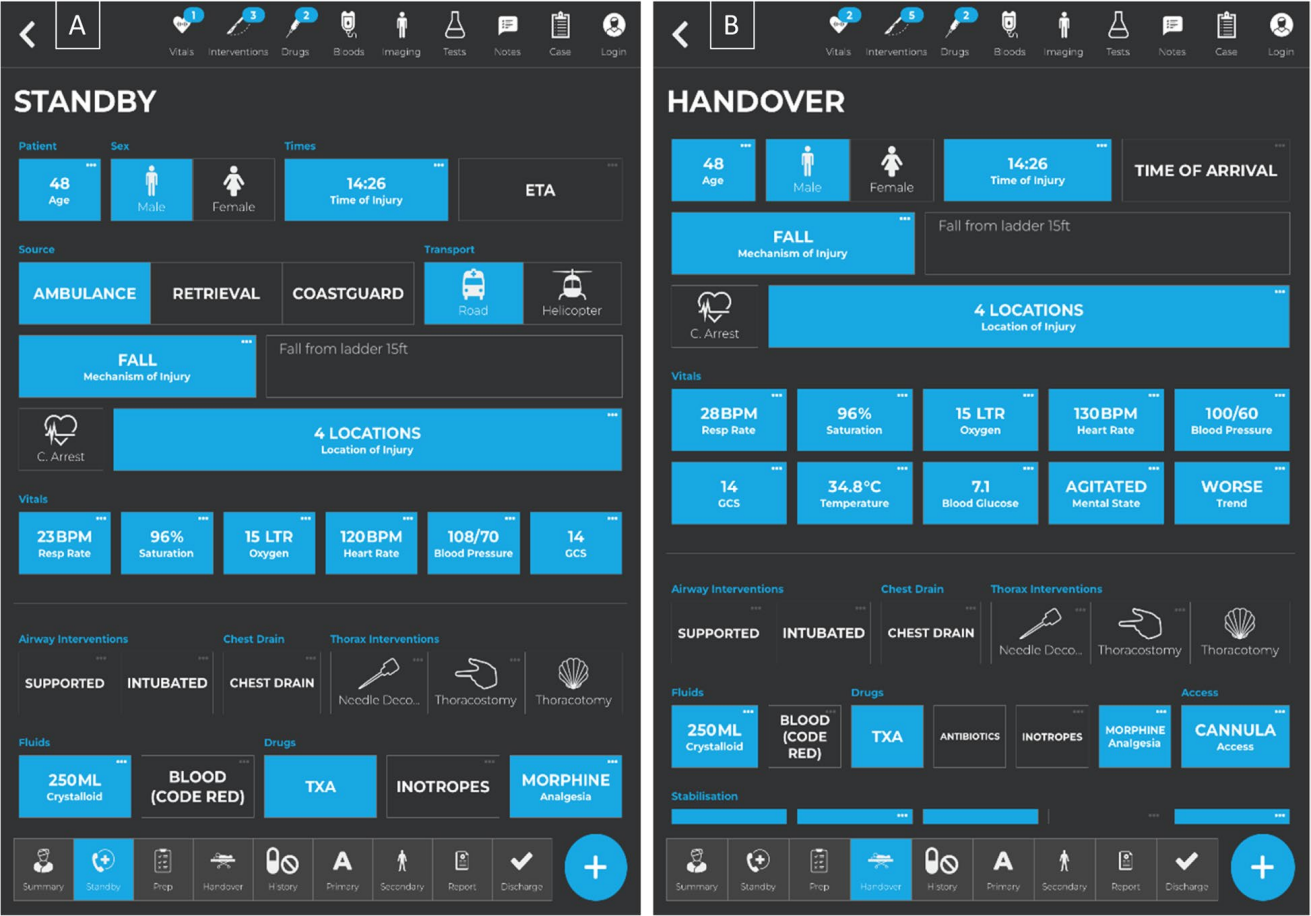
Image 1a and 1b—Standby and Handover screens from TraumaApp

Prior to assessment, the participants who used the TraumaApp were given five minutes to familiarise themselves with the ‘Standby’ and ‘Handover’ screens (Fig. 1) on departmental iPads®. Following completion of the five cases they were asked to complete a System Usability Scale (SUS) [16]. This 10-question scale was selected as the most used and reliable measure of usability [12]. Free text feedback was also collected for both the app and the paper proforma.

Data were collected on an Apple iPad® using development version 1.0.6 of the TraumaApp based on iOS (DaySix Edinburgh, UK). Data were analysed by un-paired t-test using Microsoft Excel (Version 2103 Office 365).

## Results

### Demographics

Of 50 participants, two were called away during the initial case recording and were excluded from the results. Of the 48 included participants, there were 30 Junior Doctors (62.5%), seven Consultants (14.6%), seven Nurses (14.6%), two Major Trauma Coordinators, one Acute Care Nurse Practitioner and one Student Nurse. Table 1 describes the characteristics of the participants including their familiarity with Apple operating system and personal use of digital device.

### Primary analysis

**Fig. 2:**
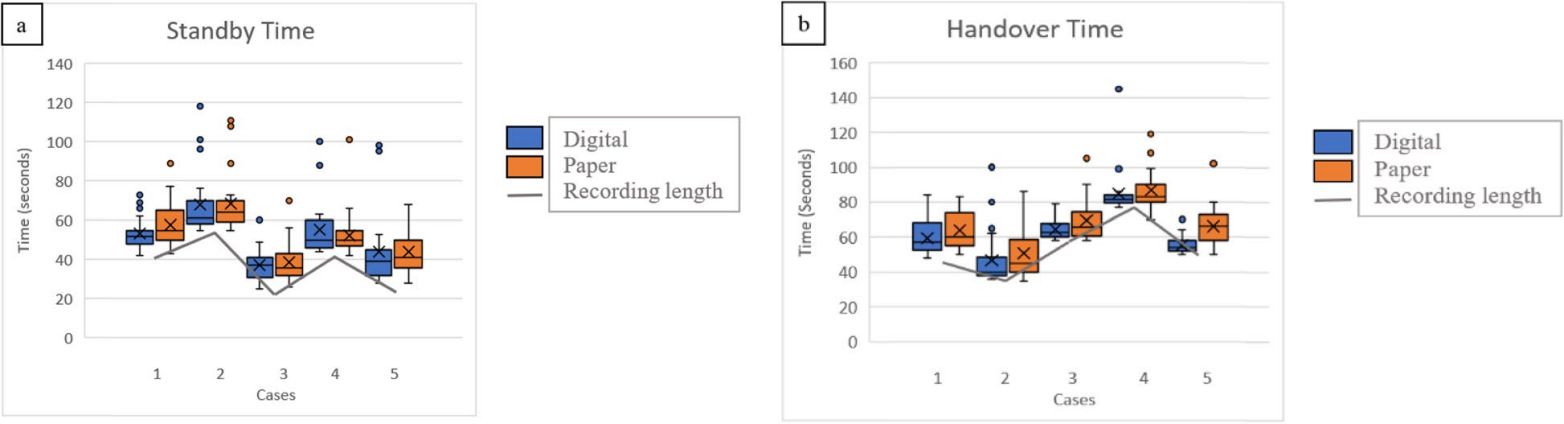
Mean time taken to input data (seconds)

Time taken to input data for each recording is displayed in Fig. 2 for digital versus paper documentation. The length of the recordings is also shown.

There was no statistically significant difference between times taken for data entry for the digital and paper documentation, apart from the Case 5 Handover (*p* < 0.05)(Table 3).

**Table 3:** Mean time taken to input data and *p*-values (rounded to 2dp)

Case Number		Digital (secs)	Paper (secs)	Significance (*p*-value)
**1**	**Standby**	54.63	57.80	0.32
	**Handover**	59.30	63.87	0.13
**2**	**Standby**	67.46	67.96	0.91
	**Handover**	46.88	50.63	0.40
**3**	**Standby**	37	38.63	0.54
	**Handover**	64.33	69.75	0.05
**4**	**Standby**	54.83	52.21	0.48
	**Handover**	84.71	86.67	0.58
**5**	**Standby**	43.75	43.83	0.98
	**Handover**	55.21	66.21	**0.0001**

**Fig. 3:**
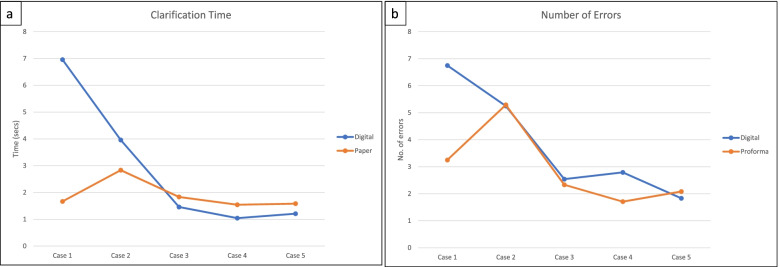
Time to clarifications (seconds) and number of errors

Figure 3 shows data for mean time needed for clarifications after listening to the recordings, together with the mean number of errors noted.

Case 1 showed a significantly higher time for clarifications and number of errors with digital data collection (*p* = 0.01 and *p* = 1.79E-05 respectively). There were no other differences between data for the app and the proforma.

#### SUS Score

**Fig. 4:**
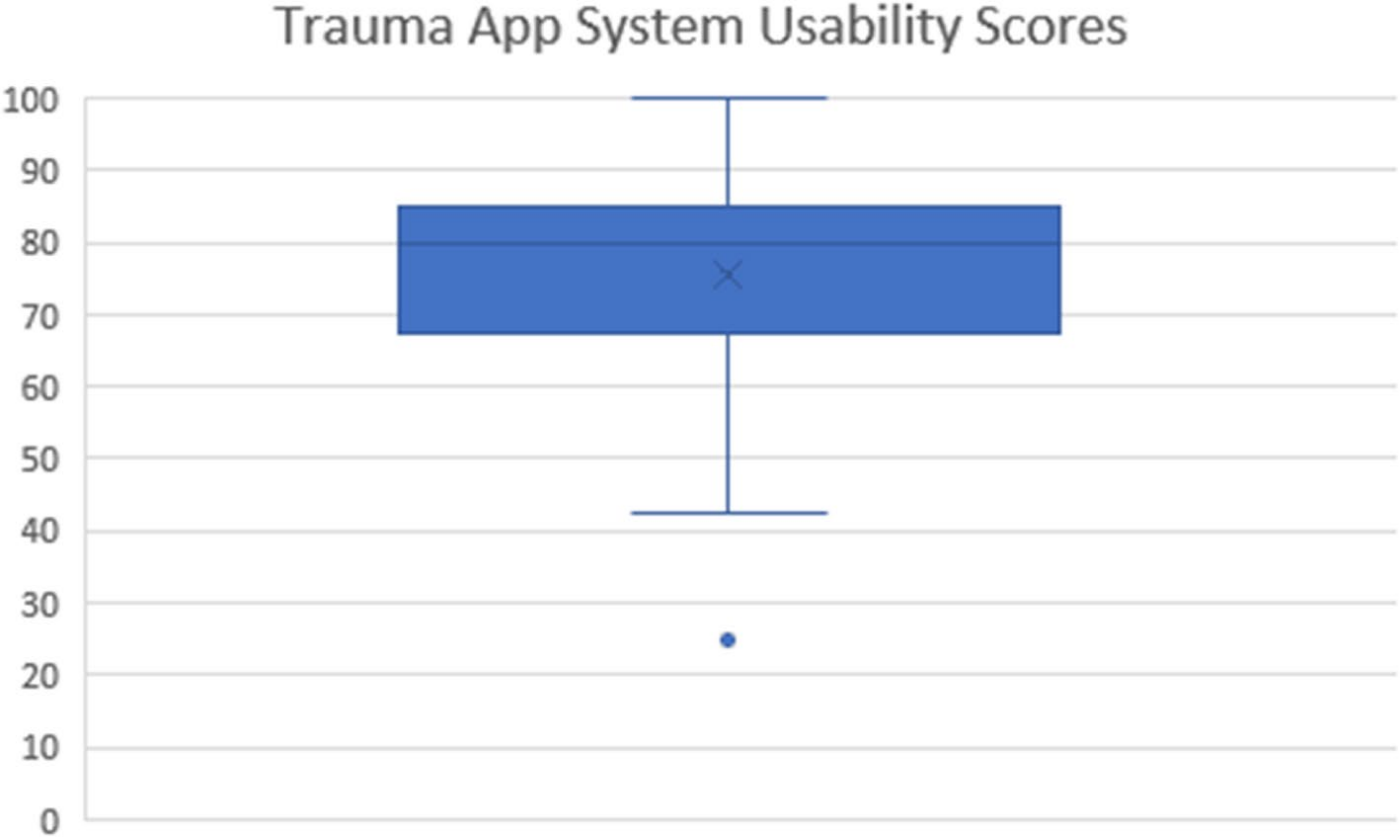
SUS scores

All participants using the TraumaApp (*n* = 24) completed a System Usability Score (SUS) questionnaire after completing the data entry. The mean SUS score for this cohort was 75 out of 100, with a standard deviation of 17 (rounded to nearest integer), as shown in Fig. 4.

### Improvement in app use/ familiarity over time

**Fig. 5:**
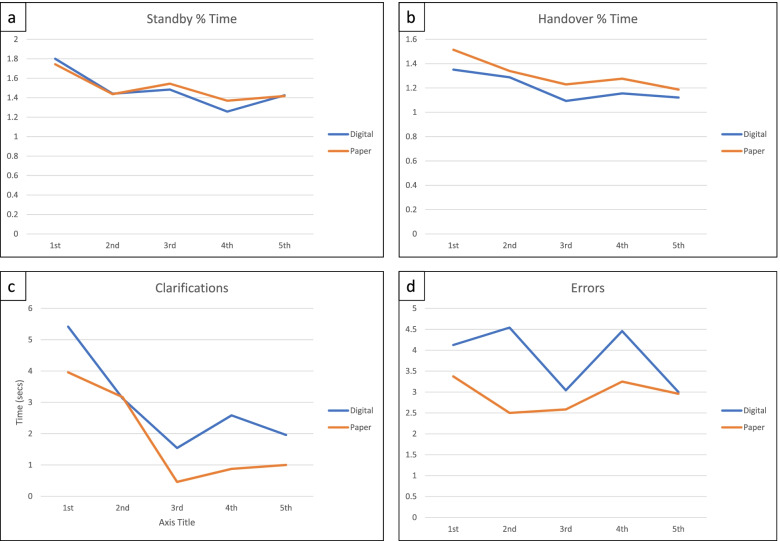
Percentage time taken to input data, clarification time and number of errors from first to final recording played.

The recorded data input times were standardised as percentages of recording duration and then re-ordered in the sequence that they were played to the participants. The mean values were compared in groups Digital and Paper, as shown in Fig. 5. There was no significant decrease in time taken over repeated use of the app or the paper proforma.

## Discussion

Early identification and management of injuries following major trauma is critical in reducing morbidity and mortality [7]. This relies on clear communication, synchronous assessment and point of care investigations. Accurate documentation allows the clinical team to have a shared understanding of the case and enhances team situational awareness, to provide effective, safe care [17]. Contemporaneous documentation is often complicated by the rapid and high volume of information-rich data in often busy chaotic environment. This is most notable during standby calls, where documented information allows for appropriate trauma call preparation, and handover at arrival to hospital, where patient condition and pre-hospital treatment are relayed to direct ongoing management [18].

This study shows equivalence between digital and paper documentation time of standby and handover. The results show that both digital data collection and use of paper proforma result in some loss of information and errors during Standby and Handover, which has been identified in previous studies [19]. The significantly higher clarification time and volume of errors in the digital cohort seen in Case 1 was deemed to be due to a high volume of information to be entered. Further work is required around training to enhance familiarity and to optimise the user interface to facilitate data entry. The System Usability Scale was developed in the 1980s to allow objective assessment of perceived usability [20]. A mean score of 68 has been internationally validated as the standard of acceptable usability [12], and the 25 participants who completed the questionnaire gave a mean score of 75.

A secondary objective to examine familiarity to data recording over time was more difficult to assess, due to the differing lengths and complexities of the recorded cases. The case orders were randomised to combat this, and the time taken to record data was calculated as a percentage of each case’s recording length, as shown in Fig. 5a and b. A general downward trend over time, seen in Fig. 5a and b, suggests development of familiarity in both digital and paper documentation. No true assessment could be made about improvement in use of the Trauma App over time, as each participant only used it for 20–25 min. Previous studies of digital system use suggests that development of familiarity with the icons and images used in an application is required to improve operating efficiency [21]. This indicates that users may benefit from instruction on the TraumaApp but the addition of iconography would potentially increase the training demand. Twenty of the 24 app users reported that they became more familiar with the app as they used it, although this is not supported by the data in Fig. 5.

### Limitations

This was a single-centre study involving a small cohort of participants. An increased number of participants may have given more power to allow identification of significant differences between digital and paper documentation. The TraumaApp continues to be refined prior to live release, so data may not be truly representative of the version used in practice. Demographic of participants is not representative of those who are likely to be scribing for trauma cases.

## Conclusion

No significant difference was identified overall in the utility of digital documentation using the TraumaApp compared to the existing paper proforma in this study. The System Usability Scale for the TraumaApp was above the internationally validated standard of acceptable usability. There was no evidence of improvement in use over time or familiarity, most likely due to the brevity of the assessment. This would benefit from further research, exploring data completeness and a potential mixed methods approach to gain further user feedback and assessment of performance and utility.

## Supplementary Information

**Additional file 1. **Transcripts of five cases.

## Data Availability

The datasets generated and/ or analysed during the current study are available from the corresponding author on reasonable request.

## Abbreviations

STN: Scottish Trauma Network
MTCs: Major Trauma Centres
QEUH: Queen Elizabeth University Hospital
STAG: Scottish Trauma Audit Group
ED: Emergency Department
SUS: System Usability Scale
