# Supplementary material for: Usability of novel major TraumaApp for digital data collection
Source: BMC Emerg Med. 2022 Mar 12;22:39. doi: 10.1186/s12873-022-00578-9 (PMC8917623; doi:10.1186/s12873-022-00578-9)
Supplement: Supplementary file 1 — Additional file 1. Transcripts of five cases. [file 12873_2022_578_MOESM1_ESM.docx]

**Appendix 1 – Transcripts of five cases**

Case 1 Transcript – Standby (41 secs)

“Standby call, Scottish Ambulance Service, via road
Hi, 25 year old male passenger involved in a road traffic collision approximately fifteen minutes ago. We think he might have a C-spine injury and he has a chest injury

At the moment he is GCS 15, his sats are 94% on 6l nasal cannula, he has a respiratory rate of 25, his heart rate is 111 and his BP is 98/45.

We have given 250ml of saline, he has had 5mg of morphine for his chest pain and we have immobilised his C-spine

ETA 10 minutes, over.”

Case 1 Transcript – Handover (43 secs)

“Now arrived in the department
Hi guys, unfortunately this gentleman has deteriorated on transfer, he dropped his sats and his blood pressure, and I felt he had absent breath sounds on his right side so he’s had a needle decompression on route, and now the pattern is that he’s improving

His saturations are now 100% on 15L trauma mask, he’s got a respiratory rate of 18, he has a heart rate of 103, his blood pressure is now 116/55, his GCS is 15, his temperature is 36.7 and his blood sugar was 4.5. I have put a venflon into his right second intercostal space

And he’s on a scoop.”

Case 2 Transcript – Standby (55 secs)

“Hi its Kevin on the retrieval service, just letting you know that we’re bringing in a 21 year old female that’s fallen from a horse, we’re not sure at what time, she certainly wasn’t wearing a helmet and she’s got an obvious head injury with a left leg injury and a left shoulder injury. We will probably be with you in 20 minutes

At the moment she has sats of 96% on a trauma mask with a respiratory rate of 12. Her heart rate and her blood pressure are okay with a heart rate of 99 and a blood pressure of 98/56, she is hower GCS 13 E3 V4 M6 repeat E3 V4 M6, we’ve given her 5mg of morphine, she has had 250 mg…ml of normal saline, we’ve splinted her… left femur and we have immobilised her C-spine

We’ll see you in about 15-20 minutes, over”

Case 2 Transcript – Handover (36 secs)

“Hi guys, right, sorry, this young girl had a little bit of a problem on the helipad and dropped her GCS and her blood pressure went down to 90/45, heart rate’s gone up to 110 and her GCS went down to 3. We decided since we were on the roof that we’d just stick in an LMA, give her 15L and use bag valve mask to ventilate her on the way down at a respiratory rate of approximately 10 …12 and decided to come straight down here.

As you can see she’s got a collar on and a femoral splint on her left leg”

Case 3 Transcript – Standby (21 secs)

“Hi its Kevin I’m on for the retrieval service. I’m going to be bringing in a 67 year old man who’s had a significant head injury, GCS 3 on the scene after he’s fallen from a cliff. He was intubated at the scene, we’ll be with you in about 20 minutes, we’re taking him in by coastguard.”

Case 3 Transcipt – Handover (58 secs)

“Hi guys this is John Smith, he’s a 67 year old chap that we were tasked to, he fell from a cliff about 45 minutes ago now, well over 3 metres. On arrival he was GCS 3 with significant signs of a head injury. He was intubated at the scene, he was a grade 1 size 9 with Ketamine, Alfentanil and Rocuronium of which I’ve just given 50mg 10 minutes ago

His heart rate now is 106, blood pressure is 95/60. He’s got two intravenous cannula, both antecubital fossas left and right, he’s got a pelvic splint on with C-spine immobilisation. We’ve given him tranexamic acid 1 gram, he’s had morphine midazolam on route, and he’s had 250ml of fluid in total”

Case 4 Transcript – Standby (41 secs)

“Hi guys I’m on for the retrieval service, we’re going to be with you in 10 minutes with a 48 year old man that’s had a blast injury to his face

On arrival he had quite severe injuries to his face so we attempted to intubate him which failed so he’s now got a surgical cric in place. We’re ventilating through that

Heart rate is 115, blood pressure is 110/60, we’re ventilating him as respiratory rate of 12 times 500. He’s got no evidence of any other injuries as far as we can see. See you in 10 minutes.”

Case 4 Transcript – Handover (76 secs)

“Hi guys this is John McKay, he’s a 48 year old guy, whilst at work today a gas cannister exploded into his face causing quite severe facial injuries.

On arrival he was sitting forward, gurgling with as you can see quite marked injuries to his face. The decision was to put him to sleep so we intubated and ventilated, or attempted to at least which was failed due to the extent of his injuries, he therefore got a surgical cric.

He was intubated with ketamine, rocuronium and alfentanil and we’ve been keeping him asleep with some midazolam and morphine on route. He’s got 2 IV access, both antecubital fossas.

We’ve immobilised his C-spine because of the extents of the head injuries but other than that we can’t see any other injuries to his body.

His heart rate on route was sitting at 96 his blood pressure has been pretty consistent around 110 systolic over 80, saturations after intubation have been 99-100% on an FiO2 of 100. We haven’t given him any tranexamic acid but he has had some cefuroxime.”

Case 5 Transcript – Standby (25 secs)

“Hi its Kevin I’m on for the retrieval service, I’m bringing you in a 17 year old female in 10 minutes who’s had a single stab wound to the chest and has undergone a thoracotomy at the scene. She had a huge pericardial effusion, cardiac massage is ongoing. We’ve given her 3 units of blood and she’s got 2 IO access. We’ll be with you in 10 minutes.”

Case 5 Transcript – Handover (50 secs)

“Hi sorry this is your 17 year old girl, we believe her name to be Gemma Smith, she has a single stab wound to her chest around 40 minutes ago. We arrived 15 minutes after the injury, she was sitting speaking to us but deteriorated pretty quickly. She had 2 thoracostomies and subsequent thoracotomy because the ultrasound showed a large pericardial tamponade and… which revealed also a wound to her right ventricle.

There is an LMA in situ and she’s getting bag valve masked with that, she’s got 2 IOs in both shoulders, she’s had three units of blood in total and some tranexamic acid.”
